# What Are the Best Practices for Nursing Care during an Earthquake? A Scoping Review

**DOI:** 10.3390/ijerph21050535

**Published:** 2024-04-25

**Authors:** Sherley Dorothie Pierre, Maíra Catharina Ramos, Helena Eri Shimizu

**Affiliations:** 1 Postgraduate Programme in Nursing, Faculty of Health Sciences, Darcy Ribeiro University Campus, University of Brasilia, Brasilia 70910-900, Brazil; sherleydorothiepierre07@gmail.com; 2 Postgraduate Programme in Public Health, Faculty of Health Sciences, Darcy Ribeiro University Campus, University of Brasilia, Brasilia 70910-900, Brazil; mairacramos@gmail.com; 3 Faculty of Health Sciences, Darcy Ribeiro University Campus, University of Brasilia, Brasilia 70910-900, Brazil

**Keywords:** nursing care, advanced nursing practice, earthquakes, review

## Abstract

Among natural disasters, earthquakes have a considerable impact and are among the ten deadliest, with an extreme impact on the healthcare sector. This study aimed to analyze the best practices in nursing care for earthquake victims. An in-depth analysis was carried out by using a scoping review, a method used in accordance with the PRISMA-ScR recommendations, to identify best nursing practice in these circumstances based on searches of eight databases: MEDLINE via PubMed; Cochrane Library; Embase; VHL; PDQ-Evidence; Scopus; ProQuest; and Google Scholar. Twenty-one studies were selected. The nursing practices identified were grouped into two distinct dimensions, each subdivided into four subcategories: (i) care practices: (a) immediate care, (b) intermediate care, (c) psychosocial care, and (d) ethical care; (ii) care management and coordination practices, which cover (a) care coordination, (b) victim care network organization, (c) teamwork, and (d) training. By analyzing these nursing practices during care and relief operations for earthquake victims, this study identified the various actions carried out, the nursing skills to be developed, and the reinforcement of these advanced practices through the systematization of nurses’ skills, in order to promote victims’ rehabilitation, minimize their suffering, and improve their quality of life during and after an earthquake.

## 1. Introduction

The World Health Organization (WHO) defines natural disasters as “earthquakes, tsunamis, volcanic eruptions, landslides and hurricanes, with lasting physical, biological and social impacts on the health, well-being and survival of individuals” [1]. Annually, natural disasters affect approximately 160 million people, killing around 90,000 people [2]. Among natural disasters, earthquakes have a considerable impacts, being classified among the ten deadliest disasters. In 2022 alone, more than 380 risks and natural disasters occurred around the world, resulting in the loss of 30,704 lives and affecting 185 million individuals [3].

When analyzing non-structural damage from earthquakes, almost all forms are classified as non-fatal injuries. Furthermore, the increased transmission of infectious diseases and subsequent outbreaks are common consequences of the primary catastrophe [4]. Added to these consequences are the displacement of populations (internally displaced people/refugees), mental health problems (including increased rates of depression), environmental changes, and an increase in vector reproduction sites, such as an increased risk of malaria [4].

Indirectly, earthquakes also affect, in the short term, the drinking water supply system, electricity, road networks, and communication, causing a shortage of pre-hospital care and difficulties in registering and tracking patients [4,5]. In the long term, earthquakes can affect health surveillance and interventions, such as immunization and vector control programs, in addition to causing disruptions to local services, resulting in a reduction in healthcare services and a possible shortage of healthcare products and equipment, especially when physical structures that provide healthcare services are affected [4].

In this scenario, it is essential to prepare nursing professionals to respond to these events, in order to reduce the negative consequences for affected populations’ health. For health systems and the provision of healthcare in these situations to be effective, nurses must have essential capabilities or skills for a quick and effective response [6].

The International Council of Nurses (ICN) noted in its 2009 Framework of Disaster Nursing Competencies the critical role these professionals play in disasters. With the largest group of engaged healthcare staff, nurses serve as first responders, screening agents and care providers, care and service coordinators, information or education providers, and, ultimately, counselors [6]. There is still no clear definition of disaster nursing in the world, but it can be understood as the systematic and flexible use of knowledge and skills related to disasters and cooperation with multidisciplinary teams, from pre-disaster to post-disaster, to reduce health risks, and life-threatening damage caused by disasters. However, there is little research evidence to guide nurses as well as nursing training programs, and the multiplicity and diversity of disasters have led to variations in the content of responses to disaster care [7,8].

This study is justified due to the need for research that studies the evidence of best care practices, particularly nursing, as they represent an important contingent of teams that work during earthquakes; therefore, they can contribute to reducing risks, damages, and deaths. Thus, the need to raise awareness among policymakers and social assistance agencies that earthquakes are a public health priority is highlighted in order to alert professionals about the conditions they will have to deal with in the event of such disasters [9]. Thus, this study aimed to analyze care practices, especially nursing, in earthquakes.

## 2. Materials and Methods

A scoping review was carried out and conducted in accordance with Preferred Reporting Items for Systematic Review and Meta Analyses extension for Scoping Review (PRISMA-ScR) recommendations [10]. To carry out this review, the following review question was considered: what are the best nursing care practices in earthquakes? To this end, the acronym PCC [11] was used, as follows: population—this does not apply to a specific population; concept—best nursing care practices; context—care during and after earthquakes.

### 2.1. Search Strategy

The search strategy included the MeSH descriptors [(“Nursing Care” OR “Patient Care Planning” OR “Nursing”) AND “Earthquakes”] and their respective synonyms. The searches were carried out in the MEDLINE via PubMed, Embase, Cochrane Library, PDQ-Evidence, Scopus, ProQuest, and the Virtual Health Library (VHL) collection databases. A search was carried out in the gray literature using Google Scholar and reference lists of included articles. The searches took place in February 2022 and were updated on 19 May 2023. The complete search strategy is available in Supplementary Materials.

### 2.2. Study/Source of Evidence Selection

All identified studies were imported into Zotero 6.0.27, where duplicates were removed. Subsequently, references were imported into Rayyan (available at <https://rayyan.ai/> [accessed on 19 May 2023]), where included studies were selected.

To select the studies, the title and abstract were read and, subsequently, the full texts. The selection in both stages was carried out by two independent researchers (SDP and MCR), with divergences defined by a third reviewer (HES), as recommended [11].

Studies that clearly addressed the best nursing practices in cases of earthquakes were considered as eligibility criteria. Review studies (systematic or not), experimental studies (randomized controlled trials, non-randomized controlled trials), quasi-experimental studies (controlled before and after studies, interrupted time series), observational studies (cohort, case–control, sectional), and qualitative studies were included. Thus, studies carried out in any location, year, or country were also considered eligible. Studies that dealt with the care practices of other professions or that did not describe in detail the best practices identified for earthquake situations were excluded. Studies published in languages other than English, French, Spanish, and Portuguese were also excluded.

### 2.3. Data Extraction, Data Analysis, and Presentation

Two researchers (SDP and MCR) were also responsible for extracting relevant data from the studies included in this scoping review from a standard table that contained information about the author (year), title, and country where the experience was reported during and after the earthquake, objective, nursing practices, method, results, and conclusion.

After the data extraction, a qualitative synthesis of the identified studies was carried out. A qualitative synthesis is an integrative technique that summarizes research results based on thematic groupings, with the aim of combining findings from multiple studies [12]. For this scoping review, grouping was based on a deductive thematic analysis, allowing for the creation of large groups of nursing practices.

## 3. Results

**Figure 1:**
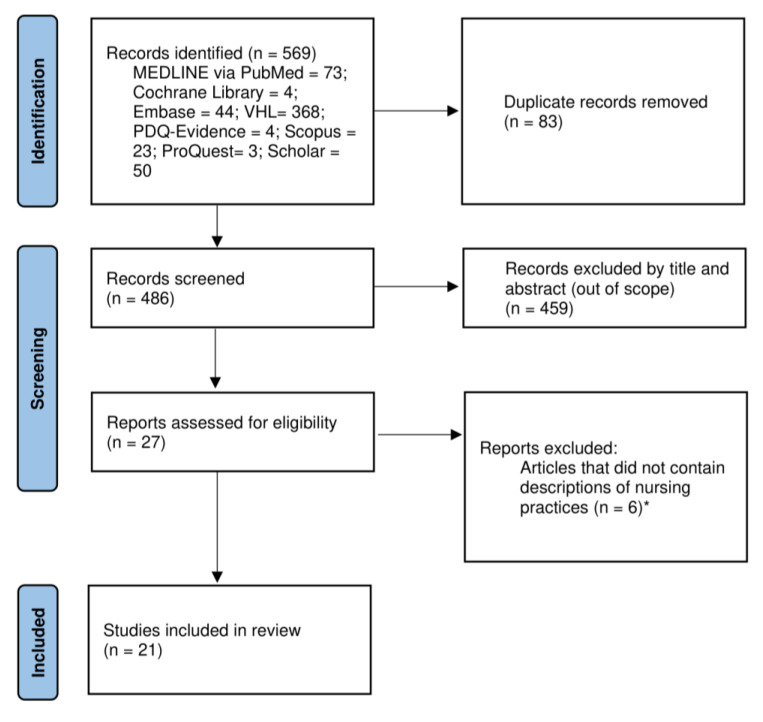
Study selection process flowchart. * The list of articles excluded with justification is available in the Supplementary Materials.

The database search and manual search identified 569 studies. After removing duplicates, the titles and abstracts of 486 publications were read, with 27 articles being selected for a full reading. Of this total, six articles did not clearly describe which nursing practice(s) brought the best results for the care of victims in earthquake situations and were therefore excluded. To compose the final sample, 21 studies [13,14,15,16,17,18,19,20,21,22,23,24,25,26,27,28,29,30,31,32,33] were selected (Figure 1).

### 3.1. Characterization of Included Articles

As for the country of origin, 19.04% were from China and Japan each. Publications from Iran and Haiti corresponded to 14.28% each. New Zealand publications were 9.52%. And publications from Indonesia, Taiwan, Turkey, Nepal, and Pakistan totaled 4.76% each.

Regarding the study method, the majority (57.14%) were qualitative studies that carried out interviews with nurses who experienced rescue and care situations for earthquake survivors. The studies that carried out focus groups totaled 9.52%, the same percentage as the studies that carried out documentary analysis and case reports. Furthermore, two studies were identified that carried out questionnaires with nurses (9.52%) and one review (4.76%). The characterization of the included studies can be identified in Table 1.

In an attempt to answer the research question, the nursing practices identified were grouped into two distinct dimensions: (1) care practices and (2) care management and coordination practices. Each dimension identified four distinct categories, as described below.

### 3.2. Care Practices

Care practices were identified in most of the included studies, demonstrating how care is an intrinsic practice in nursing. Care practices were classified into the following categories: (a) immediate care; (b) intermediate care; (c) psychosocial care; and (d) ethical care.

#### 3.2.1. Immediate Care

The first category identified among care practices was called “immediate care”. Immediate and advanced care is characterized as care provided during earthquakes, when victims are in imminent danger of death.

In order to reduce loss of life through the provision of sudden and immediate care, immediate care practices require essential skills among nurses such as CPR, intravenous insertion, airway management, prevention of hemostasis, and shock management [16,23]. Observation and monitoring skills also need to be developed [16], in order to increase nurses’ ability to screen and identify the most urgent needs [23].

Immediate care involves using protective measures to evacuate and ensure survivor safety [31], prioritizing the most serious victims [13], administering medications [19,21], treating wounds [14], and creating infection prevention and control measures [24,26,31], are crucial for victim nursing care management.

To deal with these stressful and sudden situations with their varied impacts, nurses need to develop their critical thinking skills and their ability to adapt [30]. For Wenji et al. (2015), these skills are not developed during nursing training, although they are invaluable in disaster situations with mass casualties, such as earthquakes [30].

#### 3.2.2. Intermediate Care

The second category was called “intermediate care”, which is generally provided after the victims’ condition has stabilized, such as dressings, debridement, bandages, fixed bandages, and safety bandages [23]. Proper execution of this care requires clinical assessment and judgment from nurses, especially with regard to their in-depth knowledge of wounds and infections [24]. Carrying out health examinations or tests [21], supporting pregnancy tests [21], treating wounds, assisting with nursing consultations, administering medication, and providing support in surgical interventions and hospitalizations are aspects of fundamental care that help restore victims’ physical health and remove them from potentially life-threatening situations [14,21].

As part of this care, due to the physical and psychological consequences, it is essential, in order to reduce the number of victims, to give priority to groups considered vulnerable and treat them equally, such as pregnant women, breastfeeding women, older adults, people with disabilities, people with trauma and chronic diseases, such as hypertension and diabetes as well as children [14,21,25,33], proactively carrying out home visits due to access limitations such as mobility difficulties, weakness and lack of time [21,29]. Furthermore, affectionate communication with children [20] and integration of games into care [19,20] are elements that provide a positive atmosphere, producing effective results, including adaptation to the situation.

To verify the effectiveness or expected results of the services provided, it is important to maintain daily activities [19] and to assess and monitor the activities carried out [14,19,26]. Consequently, the ability to identify factors that prevent lives from being saved remains a key element in establishing more caring relationships with others, better appreciating the importance of patient health and safety, better appreciating the value of self-esteem, and identifying and responding better to others’ needs [19].

#### 3.2.3. Psychosocial Care

“Psychosocial care” was also identified in this review. Disastrous events, especially earthquakes, can have a major psychological impact on survivors, which is why it is essential to offer support and psychological care to victims [19,27,30,31].

During a traumatic situation, such as an earthquake, interventions are necessary to limit the psychological damage suffered by victims and nurses themselves [19,31]. Psychosocial interventions include understanding nurses’ stress and pain reactions [23] and protecting their mental health and well-being through psychosocial support programs [28], since, not infrequently, they are also victims of earthquakes and are constantly worried about their own family members [20]. With this in mind, nurses from Wenchuan, China [32] emphasized the importance of psychosocial practices aimed at the nursing team, such as group discussions, to identify and report the first signs and symptoms of post-traumatic stress in victims and nurses [30].

Still on the topic of psychosocial care, the study by Nakayama et al. (2019) points to the need to maintain psychiatric patients’ treatment [20]. Due to the shortage of human resources, nurses need to be prepared to provide psychosocial care by providing medication and clinical care to these patients, in order to help them deal with the trauma suffered after experiencing an earthquake [20].

Psychosocial care practices were also identified: spiritual support as part of nursing care [19]; empathy with victims’ reactions to stress and pain so that they feel heard and supported [19]; and help for the victims to adapt to the traumatic situation, making them resilient [16,20]. Li et al. (2015) identified, among the nurses interviewed, that thinking about victims and putting them first is essential for caring for disaster survivors: “what you do there is your duty, try not to think too much about your interests. When you go there, you need to conquer yourself, be responsible and demonstrate a loving heart” [19].

#### 3.2.4. Ethical Care

Finally, the last category identified in care practices was called “ethical care”, identified in several studies [13,14,15,17,20,22,33]. Being considered a fundamental competency for nurses during disasters, ethical care demonstrates professionals’ commitment to society and the nursing profession [24].

Among the practices identified in this strategy, ethical care was considered essential in two studies included in the review [29,32]. Ethical care includes, among others, equally treating recognized vulnerable groups, such as pregnant women, breastfeeding women, older adults, people with disabilities, people suffering from trauma and chronic illnesses such as hypertension and diabetes, as well as children [15,22,24,32].

Ethical care was also identified in the study by Nicolas et al., in which the authors reported Haiti’s experience in preventing sexual violence after the 2010 earthquake, especially among vulnerable women and children with HIV/AIDS [22]. For the nurses interviewed by Nicolas et al. (2012), ethics in care can be identified by maintaining patient confidentiality and respecting the privacy of the injured [22]. Confidentiality was also identified as ethical care in the study by Rezaei et al. (2021) [24].

Respect for intercultural differences in care provision was also identified in this review as a practice that can help alleviate ethical dilemmas [29]. In the study by Susanti et al., the authors identified that respect for cultural values was an important factor for survivors: “it was very stressful to live in a new place, far from (…) where we were born, socialized, cultivated and worked. Hopefully nurses understand” [29].

Yan et al. (2015) suggest teaching nurses about “ethics”, since the area is little-considered during nursing training [31].

### 3.3. Care Management and Coordination Practices

Another practice commonly related to nursing is care management and coordination practices. This second dimension led to four categories: (a) care coordination; (b) victim care network organization; (c) teamwork; and (d) training.

#### 3.3.1. Care Coordination

Care coordination practices were also widely associated with nursing [13,15,16,18,19,21,22,24,25,26,27,28,29,30,31,32,33]. Covering diverse practices, this category included victim care process organization [26], vital for earthquake victims’ recovery.

The evidence identified also pointed out that, to save lives, it is essential to support survivor rescue management using strategies that facilitate mass casualty transport [28] and the transfer of patients from one hospital to another that has more resources available [31]. An example would be using helicopters to rescue survivors [13,29] or during missions in difficult-to-access mountainous regions [19]. In the study by Abdi et al. (2021), the authors brought the experience of nurses who worked during the earthquake in Kermanshah, Iran, and observed how the lack of knowledge about transport protocols affected the rescue of survivors: “Nurses were not familiar with patient transport protocols, flight safety and physiology [13]. They didn’t know how to transport patients by helicopter” [13].

Among the practices identified in the “care coordination” category, management and verification of hospital conditions also stand out [29], trying to identify those that had the structure to receive more patients. According to the findings, providing logistical support for the first aid operation to victims at rescue sites increases survival rates in affected communities [14,29,30].

Organizing the evacuations of affected victims to shelters constitutes an important part of the care coordination process, requiring protective measures to monitor [18] and ensure survivor safety [32]. In that case, it is necessary to assess and monitor these care and management activities [22,26], including standardized and systematic medical records [18], preferably with daily reports on victims’ situations [14].

Care coordination also includes facilitating effective communication between nurses, survivors, healthcare personnel, and emergency services [24], as well as sharing information (national and international) [16] about victims and the nurses’ own families [14]. Among these practices, Kondo et al. (2019) identified that creating lists of the telephone numbers of all medical teams involved in the rescue supported the rescue operation [18].

Adequate management and distribution of healthcare services, equipment, and teams to support rescue were also identified as important care coordination practices [13,15,21,22,29]. Abdi et al. (2021) also highlighted the need to manage uniform use, and identify and distinguish healthcare professionals to prevent people from outside the health or rescue sector from infiltrating the service [13].

The literature also pointed out that checking whether the requested medications are compatible with those provided is also an important care coordination practice [13]. The lack of supplies was a recurring point in the studies, demonstrating the need for good management of these resources [30]. In addition to management, the literature points to administering medications as essential for managing earthquake victims [27,29].

One care coordination practice identified was the implementation of screening [24] to facilitate managing mutual vulnerability and safety between professionals and victims [30]. This practice allows care to be prioritized appropriately and groups recognized as vulnerable to be treated equitably, such as pregnant women, nursing mothers, older adults, people with disabilities, people suffering from trauma and chronic illnesses such as hypertension and diabetes, as well as children [14,21,25,33].

To ensure good care management in the event of an earthquake, it is essential to develop disaster management protocols and a guide that defines the principles to be respected and the measures to be taken in such situations [16,21,27,29,30].

#### 3.3.2. Victim Care Network Organization

The second category identified was “victim care network organization”. This category encompasses the local health network organization and coordination, being a powerful element in helping earthquake survivors and victims and organizing care processes [33], hospitals, and clinics to offer them better care [13]. A study identified that the lack of coordination between victim care services meant that financial and human resources were not used efficiently, and, in many cases, the continuity of services was interrupted [13].

Equally relevant, local health service reorganization was also highlighted as essential to enable subsequent care to be provided with privacy and confidentiality to the population [16]. Among the services reported in the studies, the reorganization of prenatal and postnatal care services, delivery rooms [16], blood safety programs [22], and hospital operations were identified [18].

Finally, the results of the study by Garfield and Berryman (2011) identified that encouraging the creation of councils of voluntary organizations that provide health services can support the country’s redevelopment after an earthquake [15].

#### 3.3.3. Teamwork

Care management and coordination practices also included teamwork organization. Among the practices identified in the category, the literature points to work environment management in two studies [21,30].

Although it is not the exclusive practice of nursing professionals, healthcare team management was identified as crucial for the rapid care of victims [14,18,19,23,33]. During disasters, other professional teams start to work together with healthcare teams, such as fire teams, making the management of the professionals involved challenging [19]. Furthermore, it is necessary to pay attention to the management of volunteer professionals who arrive to support rescues [13,25]. It is necessary to include volunteers—sometimes from other countries—in rescue activities, observing the capabilities and difficulties of each of them, in order to use the workforce efficiently [13].

Teamwork management and coordination also includes organizational-level practices, such as a work team roster, so as not to create a work overload for any professional, and the adoption of uniforms as a tool for quick identification of the professionals involved, which can prevent unethical practices, such as professional identity theft [13].

As a result of good teamwork practices, evidence has shown good collaboration between professionals [19,23] and adaptation to environmental conditions [30], being crucial strategies for good coexistence and mutual respect between team members [25]. A good teamwork practice is also the implementation or coordination of actions that guarantee work teams’ physical and mental health, being emphasized by several authors [18,27,29,30,31,33].

In this regard, actions aimed at maintaining the health of workers involved in rescues were identified. A study reported the experience of “vaccination day”, highlighting the practice of promoting vaccination as important for maintaining workers’ physical health. In the study, [16] pointed out that the majority of those involved in rescues were vulnerable to infectious diseases and, eventually, these professionals could expose patients to blood and other bodily secretions. Therefore, for the safety of healthcare professionals and patients, a day was dedicated to vaccinating staff against hepatitis B [16].

In relation to maintaining the mental health of professionals involved in rescues, several studies reported actions developed during or after earthquakes [13,18,19,27,29,31,33]. According to Li et al. (2015), interview participants believed that team members should look out for each other for the good of the entire team [19]. Similarly, Wenji et al. (2014) identified that many of the interviewees were worried about their co-workers’ mental health: “He asked to come, but after he went there, his mood became very unstable, so we had to take care of him” [30]. 

#### 3.3.4. Training

The last category was called “training” and included practices aimed at training, specializing, and/or updating nursing professionals to work in natural disaster situations, especially earthquakes [16,17,18,21,23,26,30,32].

In order for nurses to deal with earthquakes and their impact on individuals, different types of training are needed [17,21,26,30,32]. The need for training in patient transport protocols; flight safety and physiology [13]; cold chain maintenance [16]; specialized care in trauma, emergencies, major surgery, sterilization, and healing equipment; as well as psychological and mental health expertise were identified in the literature [13,23]. Continuing education for the healthcare team should be promoted [17,20,26,30,32], including doctors and nurses working in NGOs [15].

Among the essential topics for nursing professionals when caring for earthquake victims, the following were identified:

- Training on hepatitis, dermatological diseases, respiratory infections in children, and health problems [16];
- Ethical issues [17,31];
- Subjects aimed at improving nurses’ management and organizational skills [32];
- Hygiene and health [16];
- Sexually transmitted diseases, such as HIV [22].

In addition to training professionals, the need for nurses to promote health to the population through different methodologies, such as leaflets and brochures aimed at the general public, was also identified [32].

Finally, the study by Garfield and Berryman (2011) identified the need for a nursing training program. The authors bring the experience of Haiti, and, for them, it is necessary to form a ladder system for nursing education, where they begin their studies as nursing assistants and, if they complete four years of training, receive a university degree as a registered nurse [15].

A critical analysis of the contents of each identified care practice was conducted based on the results, resulting in connections with dimensions of analysis, later called “care practices” and “care management and coordination practices”. Table 2 and Table 3 present a summary of nursing care practices identified in the literature for earthquakes, in dimensions: (1) care practices; and (2) care management and coordination practices, respectively.

## 4. Discussion

This study identified the scientific evidence that supports best practices in earthquakes, especially the contributions of nursing. Care practices and care management and coordination are important for earthquake responses. These practices can be characterized as advanced, given their complexity, as they involve making decisions based on clinical judgments that require specific skills to be able to provide effective and efficient healthcare with a high degree of autonomy [34]. The Pan American Health Organization (PAHO) and WHO recommend increasing the number of advanced practice nurses to develop a valid health practice capable of meeting the population’s health needs [5].

### 4.1. Nursing Care Practices in an Earthquake

The severity of the clinical condition of people affected by earthquakes requires nurses to use advanced practices [34], requiring the development of specific skills such as CPR, intubation, and bleeding control to avoid hemorrhagic shock [24,31]. Performing immediate care skills can be an opportunity to limit the loss of life and ensure victims’ recovery. Furthermore, it was reported in the Olimpio study that nurses specializing in clinical skills and judgment improve care delivery thanks to their training and practical experience [35].

After injured individuals’ clinical condition has stabilized, the care referred to in this study as intermediate care is essential for maintaining lives. Treating wounds, administering medications, performing examinations and tests, and supporting surgeries are also a group of earthquake nursing interventions. In addition to care in health camps, the study by Susanti et al. showed the importance of carrying out home visits proactively, due to physical or time constraints [29].

Extreme conditions are very difficult for both patients and nurses, so it is essential to develop adaptability in nurses so that they can deal with reality and the work environment [19]. During an earthquake, victims may be affected by psychological problems or mental disorders. Therefore, advanced psychological skills are needed, more specifically in developing an understanding of post-traumatic symptoms [19,24,27,28,31,32] as well as promoting resilience in the face of traumatic shock [9].

Ethical care is also important. It is considered that nurses have a duty to maintain patient confidentiality, which can facilitate the provision of care, but also victim safety [24]. Respect for cultural and spiritual differences can be a means of mitigating ethical dilemmas in services and ensuring patient comfort [14,27,29].

### 4.2. Nursing Care Management and Coordination Practices in an Earthquake

Among advanced nursing practices, earthquake management and coordination encompass a series of interventions designed to organize, distribute, and care for earthquake victims. To provide the best care during earthquakes, it is necessary to implement and monitor emergency planning, which may vary from one area to another according to demand [36].

The results of studies [14,16,21,29] showed that the application of an emergency protocol for responding to earthquakes produced excellent results, which can be identified by good collaboration between care providers, balanced distribution of services, and the development of practical knowledge between providers. Establishing this protocol can also greatly help nurses understand the standards of care to be followed in the event of a crisis. Furthermore, it can provide very useful tools to organize the work process and facilitate the resolution of healthcare problems in care facilities.

It is essential to prioritize equitable care for groups recognized as vulnerable, such as children, pregnant women, breastfeeding women, older adults, people with disabilities, and people suffering from trauma and chronic illnesses such as hypertension and diabetes, as these groups’ mobility and self-defense difficulties can have a negative impact on their health [14,15,22,29].

Planning the means of transport to move victims and the supply and delivery of equipment and medicines requires good logistical planning and adequate management, depending on the field of action [18]. Lack of medical equipment can increase victims’ vulnerability and create ethical dilemmas for nurses, as demonstrated by the results of several studies [23,27,29,30].

Teamwork with mutual respect is one of the fundamental elements that facilitates good collaboration between nurses and creates a pleasant work environment [14,18,19,21,23,25,26,27]. The results of the study by Susanti et al. showed the importance of carrying out home visits proactively, as the majority of the population expected not only care in health camps but also home visits due to physical or time constraints [29].

Communication is one of the management tools necessary to obtain excellent results in providing care during earthquakes [13,18] with good connections between health units, victims, and their families. People affected by a disaster need to stay in touch with their loved ones, but also continue to have access to information about the help they receive, education, care, and follow-up. All these elements can help them to return to have a better life [37]. Study results show that when communication is adapted within the care team, it promotes better transmission of information, allowing victims to receive the best care [38]. In this regard, Maret et al. report in their results that the main challenge encountered in the first hours after the occurrence of a disaster is to ensure the dissemination of clear information, reflecting affected populations’ priority needs [38].

It is important that healthcare professionals are adequately trained so that they can react appropriately in the event of a disaster [38]. For nurses to be effective in providing care, study results [13,16] recommend training in specific areas, such as patient transport protocols so that nurses know the standards to be observed when transporting victims, to avoid causing them harm. At the same time, knowledge of emergency plans and, in particular, the procedures to be followed in the event of a disaster, is an important element to master, as mastery of protocols creates certainty and a sense of safety in providing care [39].

Likewise, promoting health education on topics such as hygiene, health, and sexually transmitted diseases, such as HIV, is essential to prevent the spread of infections and diseases and protect the population and people living with HIV [16,22].

A training program for community health nurses should be established based on the results of this study [15] with the aim of preparing nurses to become more involved in earthquake care.

Encouraging ongoing training for healthcare staff, as well as doctors and nurses working in NGOs, is not only a way to strengthen healthcare providers’ capacity but also to ensure the quality of care [21,26,30,32], more specifically to improve the quality of care in matters such as hepatitis, dermatological diseases, respiratory infections in children [16].

The results of [16] showed the need to train nurses on subjects such as hepatitis, dermatological diseases, respiratory infections in children, health problems, ethical issues, and subjects aimed at improving nurses’ managerial and organizational skills, in order to guarantee adequate care in the earthquake’s worrying conditions, in addition to flight safety and physiology [13]. Likewise, the cold chain must be maintained to protect equipment that needs to be kept cold, in addition to specialized care in traumatology, emergencies, major surgery, sterilization, and healing equipment, and knowledge of psychology and mental health for effective care [16].

From a human perspective, some disasters have an impact on health, and the damage caused affects all sectors of society and the nation as a whole. If so, we need to raise awareness of the importance of the issue and commit to ensuring that hospitals and healthcare facilities are safe and robust in the face of natural disasters. Awareness and commitment are essential. This involves awareness and commitment on the part of political decisionmakers and the public as a whole [9]. In this same context, we suggest the advanced practice of nurses’ clinical and professional skills, as well as the development of personal skills, in order to care for victims more effectively and adequately, preventing and mitigating the main risks in providing care in these complex and distressing situations that are earthquakes.

It was observed that this study does not take into account nurses’ perception of care practices to be provided in earthquakes. Additionally, creating an immediate and emergency care unit and setting up care locations are good ways to allow healthcare staff to provide care in complete safety. Including nurses in the development of disaster plans will allow them to develop other interdisciplinary skills, develop their leadership capabilities, and be more effective when taking charge [38]. Better knowledge of the institution where nurses work is also important, as it can help them better manage space, i.e., optimize facilities’ capacity to better deal with any influx of victims [40].

Of the limitations of this study, it is important to consider that, although we used rigorous approaches to describe and explain how and why nursing practices work as strategies to care for and save lives in earthquake situations, the majority of included studies consisted of reviews, observational, and/or qualitative studies, we therefore attempted to describe relationships between events and outcomes (care delivery, efficiency, and quality) rather than attributing any causal effects.

## 5. Conclusions

It was found that the best nursing care practices in an earthquake involve high clinical skills and, therefore, can be characterized as advanced practices. These practices, which require specific training, save lives and/or can reduce harm to victims.

Furthermore, it was found that various management and coordination practices, in which nurses exercise leadership, guarantee adequate care for victims in an adequate time. It is also important to highlight that it is essential to develop recovery practices together with communities because the damage caused by earthquakes can last a long time.

## Figures and Tables

**Table 1:** Characterization of included studies.

Author, Year	Study Design and Method	Objective	Main Practices Identified
Abdi et al., 2021 [13]	Qualitative study. Semi-structured interviews were conducted with 16 nurses involved in providing care to those injured in the Kermanshah earthquake. After transcription, conventional content analysis was performed using the Graneheim and Landman approach.	Discuss the challenges faced by nurses in caring for victims of the Kermanshah earthquake in 2017.	Prioritize victims; Coordinate and organize regional field hospitals; Efficiently use financial and human resources; Use helicopters to rescue survivors; Check consistency between ordered medications and supplied items; Have a good command unit; Assign roles between institutions/organizations; Manage volunteers; Manage nurses’ physical and mental health; Manage uniform use, identification, and distinction for professionals; Manage internal communication; Manage material and human resources; Promote training in patient transport protocols, safety, and flight physiology.
Amat Camacho et al., 2018 [14]	Retrospective, documentary-based descriptive study. A search was carried out on PubMed and Google on International Emergency Medical Teams (I-EMT) using the STARLITE methodological principles (sampling strategy, type of study, approaches, year range, limits, inclusion and exclusions, terms used, electronic sources). Based on the results, the authors selected studies that addressed the timing and activities of I-EMT during the 2015 earthquake in Nepal and were included in the study.	Describe the characteristics, timing, and activities carried out by I-EMTs deployed in Nepal following the 2015 earthquake and assess their adherence to the WHO I-EMT	Treat wounds, consultation, admission, and surgery; Manage teams; Prioritize vulnerable groups (older adults, pregnant women, patients with chronic illnesses, major trauma cases); Make it easier for nurses to share information and obtain information about their own families; Facilitate first aid to victims; Manage daily reports Manage language barriers; Manage national treatment protocols and I-EMT treatment protocols.
Garfield and Berryman, 2011 [15]	Qualitative descriptive study that sought to describe the situation of nursing education in Haiti.	Not reported	Prioritize care for vulnerable groups (older adults, pregnant women, patients with chronic illnesses); Prepare doctors and nurses who work in non-governmental organizations (NGOs); Enable the implementation of a nursing education program; Promote the creation of boards of voluntary organizations that provide health services.
Gulzar et al., 2012 [16]	Qualitative evaluative study, using the planning cycle structure as a theoretical basis. Focus groups and in-depth interviews were carried out and analyzed (content analysis) and categorized thematically.	Describe the experience of interventions carried out by community health nurses through a guided framework (assessment, planning, implementation, and assessment components)	Establish disaster management protocols; Facilitate information sharing (national and international); Reorganize prenatal and postnatal services and delivery rooms; Vaccinate nurses against hepatitis.
			Provide continuing education in areas such as hepatitis, skin problems, respiratory infections in children, and health problems; Train on cold chain maintenance; Offer educational modules on hygiene and health.
Kalanlar et al., 2021 [17]	A review was carried out in five databases (CINAHL, MEDLINE, PubMed, Scopus, and Web of Science), using the keywords “earthquake” and “nursing” for studies published between 2010 and 2020. Of the 665 articles identified, 19 were included in the review.	Establish a general framework of evidence on earthquakes and nursing and develop recommendations for future studies in this field.	Respect intercultural differences in care provision; Resolve conflicts and ethical dilemmas; Promote psychosocial support programs to protect nurses’ health and well-being; Facilitate transportation of large numbers of victims; Manage emergencies and interventions in psychological crises; Facilitate obtaining information about victims and families, including from nurses themselves; Promote continuing education; Provide training programs, including assessing ethical issues related to disasters.
Kondo et al., 2019 [18]	Qualitative descriptive study. All communication records made in July 2016 in Kumamoto, Japan were assessed. After reading the records, the main recorded events were selected.	Identify improvements in disaster medical operations from the 2016 Kumamoto earthquake (Kumamoto prefecture, Japan) and extract further lessons learned to prepare for large expected future earthquakes.	Deploy medical rescue services and teams; Manage teams; Check the status of disaster-affected hospitals using the Emergency Medical Information System; Monitor evacuation shelters; Watch hospital operations; Coordinate the transfer of patients admitted to damaged hospitals; Use helicopters to rescue survivors; Provide healthcare in evacuation shelters; Provide healthcare at the rescue site and provide logistical support; Ensure physical and mental health conditions for teams; Share information between emergency departments (national and international); Create phone number lists for all disaster relief medical teams; Develop a standardized system for maintaining medical records.
Li et al., 2015 [19]	Qualitative study that carried out in-depth interviews with 15 nurses from five different hospitals. The interviews were transcribed and analyzed according to Grounded Theory as a theoretical approach.	Explore Chinese nurses’ earthquake experiences and develop a substantive theory of seismic disaster nursing that will help inform the future development of disaster nursing education.	Develop critical thinking and adaptability; Facilitate management of mutual vulnerability and safety between professionals and victims; Facilitate good collaboration between teams; Facilitate first aid, professional psychological trauma counseling, and psychological support; Facilitate specialized training in trauma, emergency, major surgery, sterilization, and wound healing equipment as well as psychological and mental health knowledge.
Nakayama et al., 2019 [20]	Qualitative descriptive study. A Japanese methodology called Katarai (a form of group interview) was used, consisting of 11 nurses. After the Katarai, two in-depth interviews were carried out with head nurses who worked during the earthquake.	Describe the experiences of nurses working in a psychiatric hospital in the Fukushima prefecture during the Great East Japan Earthquake and explore what sustained nurses while working in the damaged hospital.	Assist psychiatric patients; Use protective measures to evacuate and ensure survivor safety; Use infection prevention and control measures; Resolve conflicts and ethical dilemmas; Facilitate the transfer of patients from one hospital to another; Manage human waste and other waste.
Nasrabadi et al., 2003 [21]	Qualitative study carried out with 13 participating nurses. Data were obtained through serial semi-structured interviews and analyzed using the latent content method.	Explore the experiences of Iranian registered nurses in disaster relief in the 2003 Bam earthquake in Iran.	Establish disaster management protocols; Facilitate organization in the workplace; Promote continuing education; Facilitate training programs.
Nicholas et al., 2012 [22]	Case report on pediatric care in Haiti after the 2010 earthquake.	Discuss the complex interplay between an environmental emergency and the increasing risk factors and human rights issues for the pediatric population in Haiti.	Prevent mother-to-child transmission of HIV through education; Prevent sexual violence, especially among vulnerable women and children with HIV/AIDS; Treat sexually transmitted infections; Prioritize care for vulnerable groups (children, older adults, pregnant women, patients with chronic diseases including HIV, major cases of trauma); Facilitate adherence to blood safety programs.
Richardson et al., 2013 [23]	Qualitative study that conducted interviews with nurses who worked in the 2010 earthquake in New Zealand	Describe the impact of the Canterbury, New Zealand earthquakes on Christchurch Hospital and emergency nurses’ experiences during this period.	Distribute services and equipment to healthcare teams; Facilitate good collaboration between teams; Use informal communication (such as telephone contact, television news, and the Internet) to understand and report events as they are presented; Support the implementation of coordinated emergency plans, with frequent review, practice, and education; Perform patient tracking and keep clinical documentation up to date.
Rezaei et al., 2020 [24]	Qualitative descriptive study that carried out semi-structured interviews with 16 nurses involved in providing care to those injured in the earthquake in Kermanshah, Iran. Data were analyzed using the Graneheim and Lundman approach.	Identify professional skills needed by nurses to provide care to those injured by the earthquake.	Develop a sense of observation and monitoring; Develop skills in cardiopulmonary resuscitation (CPR), prevention of hemostasis, dressings, safety, manual handling and emergency management, intravenous insertion, and observation, monitoring, and screening of victims; Maintain patient confidentiality; Help nurses to be creative in providing care (soft skills); Facilitate effective communication between nurses, survivors, and the healthcare team; Facilitate adaptation to the traumatic situation.
Sato et al., 2015 [25]	Qualitative study. Semi-structured interviews were carried out with nurses who had worked during the earthquake in Japan in 2011. After transcription, content analysis was carried out according to the Hammersley and Atkinson (2007) framework.	Describe the experiences of a local government public health nurse who worked in an area affected by the Great East Japan Earthquake.	Manage volunteers; Value mutual respect; Facilitate good internal communication.
Scrymgeour et al., 2020 [26]	Pluralistic qualitative research and inductive thematic analysis were carried out. Fifteen interviews were carried out with nurses who worked in hospitals and institutions for older adults during earthquakes between 2010 and 2015 in New Zealand and Australia. Data analysis used the methodological precepts of Braun and Clarke (2006), in which inductive coding was carried out by the researcher.	Explore the factors that influence nurses’ resilience and adaptive capacity during a critical incident caused by a natural disaster.	Generate responsibility of the nursing team towards families and survivors; Promote continuing education; Coordinate patient care; Ensure patient and team safety; Develop personal skills.
Shih et al., 2002 [27]	Qualitative descriptive study. Semi-structured interviews were carried out with five highly experienced nurses. After transcription, content analysis was carried out.	Compare the impacts of rescue experiences on Taiwanese nurses and nurses who worked as first responders after the September 21 earthquake.	Help identify factors that impede the provision of vital care; Monitor the population’s health status; Advise play therapy for children; Detect psychological problems; Carry out psychosocial interventions after the earthquake; Maintain daily activities; Assess and manage health problems; Provide medicines; Treat wounds; Provide spiritual care; Establish disaster management protocols; Assess physical and mental health conditions for teams; Facilitate healthcare missions in mountainous regions.
Sloand et al., 2012 [28]	Qualitative descriptive study. Conducted in-depth interviews with 12 volunteer nurses who worked during the Haitian earthquake in 2010. The interviews were transcribed and analyzed with the support of NVivo9.	Explore the experiences of volunteer nurses caring for children following the January 2010 earthquake in Haiti.	Communicate affectionately with children; Incorporate games into childcare; Promote resilience to traumatic shocks.
Susanti et al., 2019 [29]	Qualitative descriptive study. Three focus groups were held with 21 survivors and in-depth interviews with three community leaders were held. After transcription, content analysis was carried out using the theoretical precepts of Graneheim and Lundman (2004).	Explore survivors’ expectations of disaster nurses.	Carry out health exams or tests; Administer medications; Support pregnancy testing; Proactively carry out home visits; Equitably treat groups recognized as vulnerable, such as pregnant women, breastfeeding women, older adults, people with disabilities, people suffering from trauma and chronic illnesses, such as hypertension and diabetes, and children; Respect and integrate cultural values in the provision of care; Distribute services and equipment to healthcare teams; Establish disaster management protocols; Assess physical and mental health conditions for teams; Assess and monitor the activities carried out.
Wenji et al., 2014 [30]	Qualitative study that conducted interviews with 12 nurses in China. The interviews were transcribed and analyzed in light of the theoretical framework of narrative methods.	Describe the experiences of Chinese nurses who worked in disaster relief after the Wenchuan and Yushu earthquakes and their views on future disaster nursing education/training programs.	Establish disaster management protocols; Distribute teams and services; Ensure minimum physical and mental health conditions for work teams; Facilitate organization in the workplace; Help nurses adapt to environmental conditions; Support healthcare professionals in the field of mental health; Manage resources (water, food) and medicines; Promote continuing education.
Yan et al., 2015 [31]	Descriptive study that applied a questionnaire to 38 Chinese hospitals, obtained 89 valid and analyzed responses. The means and standard deviation of the quantitative data from the questionnaire were calculated using SPSS 20.0. Qualitative data were analyzed by content analysis using the Holloway and Wheeler (2013) framework.	Explore the skills, knowledge, and attitudes required by registered nurses from across China who worked after three major earthquakes to try to determine future disaster nursing education requirements.	Develop skills in hemorrhage control, CPR, airway management, shock management, debridement, dressings, bandages, bandage fixation, and safety; Understand the stress and pain reactions of the nursing team; Teach ethics to nurses.
Yang et al., 2010 [32]	Qualitative study. Semi-structured interviews were carried out with 10 nurses. The interviews were transcribed and analyzed according to Gadamer’s philosophical hermeneutics.	Provide an understanding of how Chinese nurses acted in response to the 2008 Wenchuan earthquake.	Develop nurses’ assessment and clinical judgment skills, especially in terms of in-depth knowledge about wounds and infections; Facilitate screening and identify the most urgent needs; Discuss in a group to identify and report early signs and symptoms of post-traumatic stress disorder; Promote training sessions; Hand over information leaflets; Promote continuing education; Promote the improvement of nurses’ managerial and organizational skills.
Yokoyama et al., 2014 [33]	Quantitative descriptive study. A questionnaire was sent between December 2012 and January 2013 to nurses who worked during the Great East Japan Earthquake. A total of 1640 questionnaires were received. Quantitative variables were statistically analyzed using SPSS 20.0. For qualitative variables (subjective well-being, bad mood, worsening sleep status, and intense fatigue), forced-entry multiple logistic regression analysis was performed to identify factors associated with nurses’ health status.	Document the actual activities carried out by public health nurses during their discharge and their health status during and after dispatch to the three prefectures most severely affected by the earthquake.	Enable consultations at evacuation centers; Use infection prevention and control measures; Organize service processes; Manage work teams; Ensure physical and mental health conditions for work teams; Assess and monitor the activities carried out.

**Table 2:** Summary of nursing care practices in the event of an earthquake in dimension “care practices”.

Thematic Categories	Nursing Care Practices in an Earthquake
Care practices	Prioritize victims [13] and proactively carry out home visits [29]; Maintain daily activities [27], treat wounds, carry out nursing consultations, medication, exams and admission, and surgery support [14,27,29]; Respect and integrate intercultural differences and values in the provision of care [17,29]; Develop critical thinking and adaptability [19]; Carry out screening [32] and equitably treat groups recognized as vulnerable, such as pregnant women, breastfeeding women, older adults, people with disabilities and people suffering from trauma and chronic diseases, such as hypertension and diabetes, and children [29]; Support psychological care for earthquake victims, carrying out psychosocial interventions when necessary [20,27]; Use protective measures to evacuate and ensure survivor safety [20]; Use infection prevention and control measures [20,33], including controlling mother-to-child transmission of HIV [22]; Prevent sexual violence, especially among vulnerable women and children with HIV/AIDS [22]; Treat sexually transmitted infections [22]; Develop a sense of observation and monitoring [24]; Develop skills such as CPR, prevention of hemostasis, dressings, safety, manual handling and emergency management, intravenous insertion, observation, monitoring and screening of victims [24], airway management, shock management, debridement, dressings, bandages, bandage fixation and safety [31], nurses’ assessment, and clinical judgment, especially in terms of in-depth knowledge about wounds and infections [32]; Maintain patient confidentiality [24]; Help identify factors that impede the provision of vital care [27]; Adapt practices to care for children, carrying out clear communication and incorporating playful therapies and games into the act of care [27,28] ; Provide spiritual care [27]; Promote resilience to traumatic shocks [28], acting with empathy to stress and pain reactions [31] and facilitating adaptation to the traumatic situation [24]; Value mutual respect [25]; Promote creative solutions in the provision of care [24].

**Table 3:** Summary of nursing care practices in the event of an earthquake in dimension “care management”.

Thematic Categories	Nursing Care Management and Coordination Practices in an Earthquake
Care management and coordination practices	Coordinate and organize the local care network (hospitals, clinics, etc.) [13] and facilitate communication with the organizational network (institutions, organizations, etc.) [13], assigning roles and responsibilities between each service point; Establish and manage disaster management protocols [16,21,27,29,30]; Check the status of disaster-affected hospitals using the Emergency Medical Information System [18], facilitating the transfer of patients from one hospital to another [18,20]; Monitor evacuation shelters [18], providing healthcare at sites whenever necessary to facilitate access to healthcare for the affected population [18]; Support survivor rescue management, using strategies to facilitate the transport of large numbers of victims [17], such as helicopters [13], or in missions in mountainous and hard access [27]; Provide logistical support [18] to the first aid operation for victims at the rescue site [14,19]; Enable consultations in evacuation centers [33]; Distribute services, equipment, and healthcare teams that will support the rescue [18,23,29,30]; Assess and manage population health problems [27]; Resolving conflicts and ethical dilemmas [17,20]; Assess and monitor the care and management activities carried out [29,33]; Manage the work environment at the organizational-level scale, [21,29], healthcare teams [14,18,33] including volunteer professionals [13,25] use of uniforms, identification of professionals [13], and communications [13,25], facilitating good collaboration between teams [19,23] and adaptation to environmental conditions [30]; Manage [13] and promote psychosocial support programs to protect nurses’ health and well-being [17,30], ensuring physical and mental health conditions for teams [18,27,29,30,33] and facilitating professional psychological counseling [19]; Promote work team vaccination [16]; Promote group discussions to identify and report early signs and symptoms of post-traumatic stress disorder [32]; Manage emergencies and interventions in psychological crises [17]; Generate responsibility of the nursing team towards families and survivors [26]; Manage and efficiently use financial and human resources [13,30], checking the consistency between medicines requested and items provided [13,30]; Manage human waste and other waste [20]; Implement prioritization of care for vulnerable groups (children, older adults, pregnant women, and patients with chronic diseases, including HIV, major cases of trauma, among others) [14,15,22]; Organize victim care processes [33], including standardized and systematic medical record management [18], preferably preparing daily reports on victims [14]; Facilitate information sharing (national and international) [16] about victims, including information about their own families [14,17]; Facilitate effective communication between nurses, survivors, healthcare staff [24] and emergency departments (national and international) [18]; Facilitate managing mutual vulnerability and safety between professionals and victims [19]; Promote and facilitate training [21,32] on patient transport protocols, flight safety and physiology [13], cold chain maintenance [16], specialized care in trauma, emergency, major surgery, sterilization, and wound healing equipment as well as psychological and mental health knowledge [19]; Promote healthcare team’s continuing education [17,21,26,30,32], including doctors and nurses working in NGOs [15], on topics such as hepatitis, dermatological diseases, respiratory infections in children, health problems [16], ethical issues [17,31], and in themes for improving nurses’ managerial and organizational skills [32]; Promote health education on topics such as hygiene and health [16] and sexually transmitted diseases, such as HIV [22], using different methodologies, such as using leaflets and folders for the population [32]; Enable the implementation of a community health nursing education program [15]; Promote the creation of boards of voluntary organizations that provide health services [15]; Reorganize local health services, such as prenatal and postnatal services and delivery rooms [16], blood safety programs [22] and hospital operations [18]; Manage language barriers [14]; Create phone number lists for all disaster relief medical teams [18].

## Data Availability

Not applicable.

## Supplementary Materials

The following supporting information can be downloaded at https://www.mdpi.com/article/10.3390/ijerph21050535/s1, A: Search strategy and B: Articles excluded after complete reading, with justification [41,42,43,44,45,46].
