# Supplementary material for: What Are the Best Practices for Nursing Care during an Earthquake? A Scoping Review
Source: Int J Environ Res Public Health. 2024 Apr 25;21(5):535. doi: 10.3390/ijerph21050535 (PMC11120831; doi:10.3390/ijerph21050535)
Supplement: Supplementary file 1 [file ijerph-21-00535-s001.zip › ijerph-2812274-supplementary.pdf]

SUPPLEMENTARY MATERIAL

A – Search strategy

#1 MeSH descriptor: [Nursing Care]

#2 (Care, Nursing): ti, ab, kw

#3 (Management, Nursing Care): ti, ab, kw

#4 (Nursing Care Management): ti, ab, kw

#5 MeSH descriptor: [Patient Care Planning]

#6 (Care Planning, Patient): ti, ab, kw

#7 (Planning, Patient Care): ti, ab, kw

#8 (Nursing Care Plans): ti, ab, kw

#9 (Care Plan, Nursing): ti, ab, kw

#10 (Care Plans, Nursing): ti, ab, kw

#11 (Nursing Care Plan): ti, ab, kw

#12 (Plan, Nursing Care): ti, ab, kw

#13 (Plans, Nursing Care): ti, ab, kw

#14 (Goals of Care): ti, ab, kw

#15 (Care Goal): ti, ab, kw

#16 (Care Goals): ti, ab, kw

#17 (Nursing Care Plan): ti, ab, kw

#18 MeSH descriptor: [Nursing]

#19 (Nursing): ti, ab, kw

#20 MeSH descriptor: [Earthquakes]

#21 (Earthquake): ti, ab, kw

#22 #1 OR #2 OR #3 OR #4 OR #5 OR #6 OR #7 OR #8 OR #9 OR #10 OR #11 OR #12 OR #13 OR #14 OR #15 OR #16 OR #17 OR #18 OR #19

#23 #20 OR #21

#24 #22 AND #23

B – Articles excluded after complete reading, with justification

Articles that did not describe nursing practices in the context of earthquakes:

Kanbara, S., Lee, H. J,. Ngatu, N., Takezaki, K., Yamada, S., Nakayama, Y., et al. Information and response shortfall in shelters after the Earthquake in Kumamoto: The nursing perspective. *Heal Emerg Disaster Nurs* **2017**, 4: 74–9.

Ketchie, K., Breuilly, E. Our experience in earthquake-ravaged Haiti: two nurses deployed with a disaster medical assistance team. *J Emerg Nurs* **2010**, 36: 492–6.

Moitinho de Almeida, M., Schlüter, B.-S., van Loenhout, J. A. F., Thapa, S. S., Kumar, K. C., Singh, R., et al. Changes in patient admissions after the 2015 Earthquake: a tertiary hospital-based study in Kathmandu, Nepal. *Sci Rep* **2020**, 10: 4956.

Prasetiyawan, V. E., Maramis, A., Keliat, B. A. Mental health model of care programs after the tsunami in Aceh, Indonesia. *Int Rev Psychiatry* **2006**, 18: 559-562.

Richardson, S. K., Richardson, A., Trip, H., Tabakakis, K., Josland, H., Maskill, V., Dolan, B., Hickmott, B., Houston, G., Cowan, L., McKay, L. The impact of a natural disaster: under- and postgraduate nursing education following the Canterbury, New Zealand, earthquake experiences. *Higher Education Research & Development* **2015**, 34: 986–1000.

Sato, H., Techasrivichien, T., Omori, A., Ono-Kihara, M., Kihara, M. Psychosocial Consequences Among Nurses in the Affected Area of the Great East Japan Earthquake of 2011 and the Fukushima Complex Disaster: A Qualitative Study. *Disaster Med Public Health Prep* **2019**, 13: 519-526.
